# Reconciling competing values placed upon goose populations: The evolution of and experiences from the Islay Sustainable Goose Management Strategy

**DOI:** 10.1007/s13280-016-0880-8

**Published:** 2017-02-18

**Authors:** Rae McKenzie, Jessica M. Shaw

**Affiliations:** 1 Scottish Natural Heritage, Main Street, Bowmore, Isle of Islay PA43 7JX UK; 2 Scottish Natural Heritage, Battleby, Redgorton, Perth, PH1 3EW UK

**Keywords:** Adaptive management, Barnacle goose, Conflict resolution, Greenland white-fronted goose, Islay

## Abstract

The Scottish island of Islay hosts 45 000 barnacle geese *Branta leucopsis* (56% of the Greenland barnacle goose population, plus those passing through on migration), 5000 Greenland white-fronted geese *Anser albifrons flavirostris* (up to 30% of the world population) and 2500 greylag geese *Anser anser*, most of which feed on 9000 ha of grassland. The financial impacts of estimated agricultural damage have risen greatly over the past 20 years due to increasing goose numbers and higher farming costs. Mechanisms implemented to resolve conflict over time are reviewed for their effectiveness. Emphasis is placed on coordinating the implementation of strategic national conflict resolution at a local scale where the relative pressure from internationally important concentrations of geese on agriculture is acute. Despite the “local” nature of this problem, the benefit from the experience of decades of attempted conflict resolution and the effectiveness of existing programmes can contribute much to the regional and flyway dimensions of this international issue.

## Introduction

Migratory geese have flourished on Islay, an island off the west coast of Scotland, since the early 1980s due to the availability of high quality feeding, undisturbed roosts and the protection afforded to them under European and UK law. This has brought them into increasing conflict with agricultural interests.

In this case study, we give a historical overview of goose management on Islay and describe how management actions and attempts to resolve the conflict between conservation and agricultural interests over the years have led to the development of the current Islay Sustainable Goose Management Strategy. We describe the considerations and adaptive management approach of this ten-year Strategy, and whilst it only recently began in 2014, discuss its early days.

## Background

### Study site

**Fig. 1:**
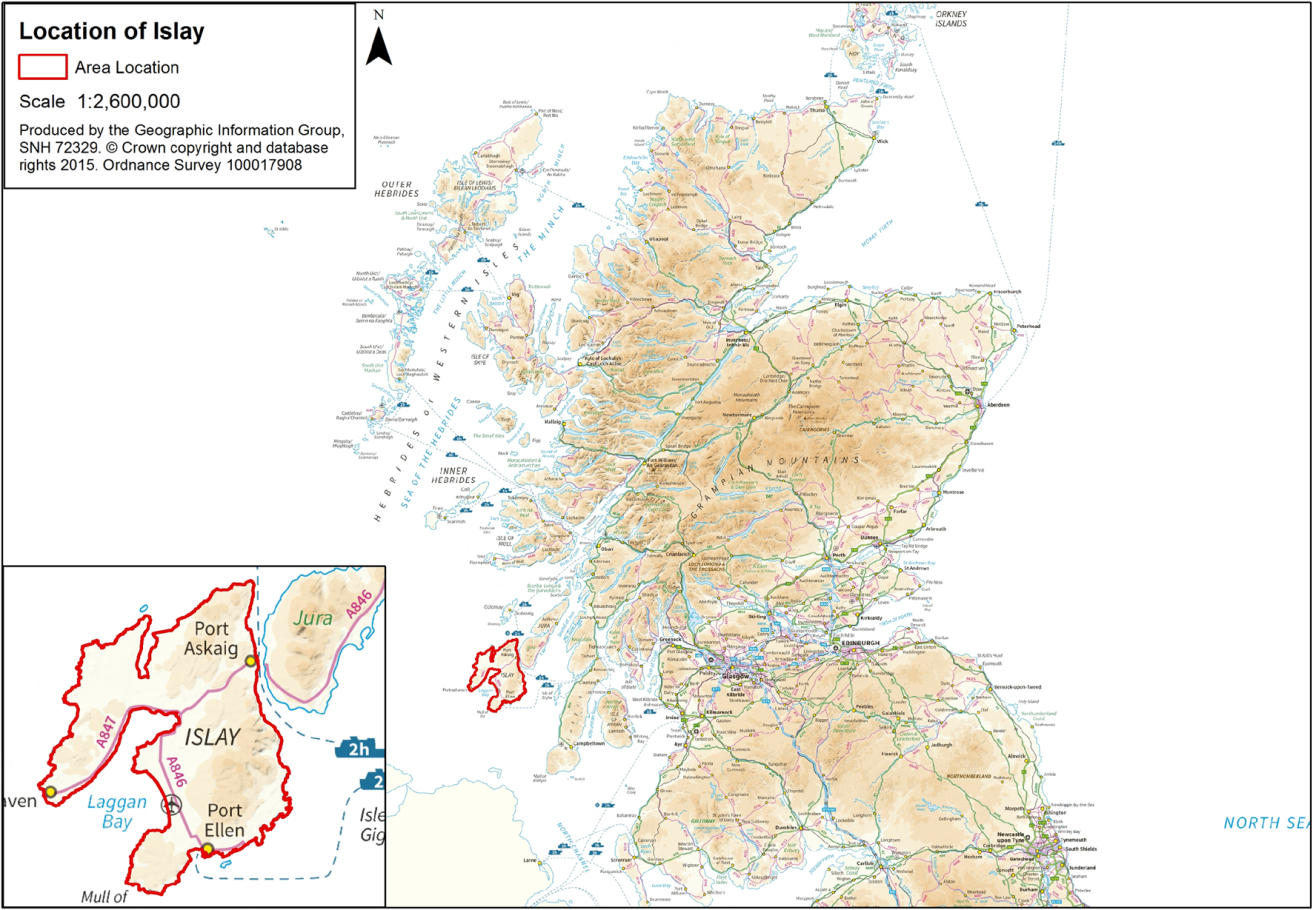
Map of Scotland showing the location of Islay

The island of Islay (Fig. 1) covers an area of around 620 square kilometres. It supports a mixture of habitats including large expanses of peatland, saltmarsh at the heads of two sea lochs and good quality agricultural ground suitable for livestock production. It has a relatively mild oceanic climate, with mean January temperatures of 2.6–7.8 °C (which allows grass growth during winter) and relatively high annual rainfall of around 1280 mm (Met Office 2016).

Agriculture is a key industry; much of the 55 000 ha of farmed land (mainly rough grazing) is used for cattle and sheep production, although some arable cultivation supplies barley to local distilleries. There are approximately 130 agricultural units, made up of full-time and part-time farmers and crofters (small scale farmers) supporting in excess of 100 full-time equivalent jobs. Just over 9000 ha of grassland is farmed, which currently supports over 5000 suckler cows and over 20 000 breeding ewes. Geese generally feed on this good quality rotational grassland, and large numbers result in a high level of damage to the agricultural economy of the island (Percival and Houston 1992; Frame 1996; Bevan 2012). There is also one dairy farm on the island, which supplies milk locally. The total income from farming and related activities on Islay is currently in the region of €12–13 m per annum (calculated using Single Farm Payment and Less Favoured Area scheme data, livestock sales figures and local knowledge from the Islay branch of the National Farmers Union of Scotland (NFUS)). Geese also attract visitors to Islay during the winter, resulting in important financial contributions to the wider island economy (Rayment et al. 1998; MacMillan and Leader-Williams 2008).

### Geese on Islay

**Fig. 2:**
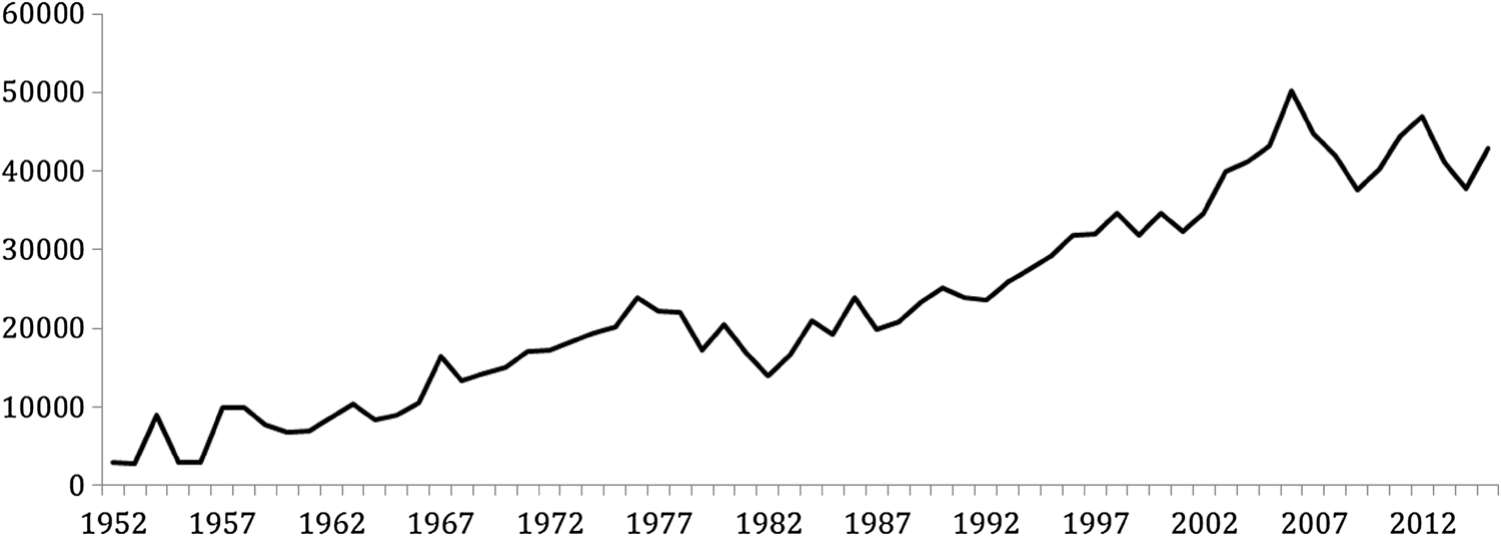
The winter mean number of barnacle geese from the Greenland population on Islay 1952–2015 (WWT and SNH count records)

There are two main wintering species with one, the barnacle goose *Branta leucopsis* (from the Greenland breeding population) currently flourishing and the other, the Greenland white-fronted goose *Anser albifrons flavirostris* currently in decline—both are listed on Annex 1 of the European Commission (EC) Birds Directive. Neither breed in Scotland. The average numbers of barnacle geese wintering on Islay rose from c. 3000 in 1952 to a peak of just under 50 000 in 2005–2006 (Fig. 2) (Mitchell and Hall 2013). Numbers have fluctuated over recent years; there was no significant growth in the Islay counts between the last two population censuses in 2008 and 2013, and recent analysis suggests that the trend has now levelled off (Hilton et al. 2014, Fig. 2). Greenland barnacle geese are legal quarry in Iceland on autumn migration, and have been subject to shooting on Islay, under licence, since 2000 (described further below).

**Fig. 3:**
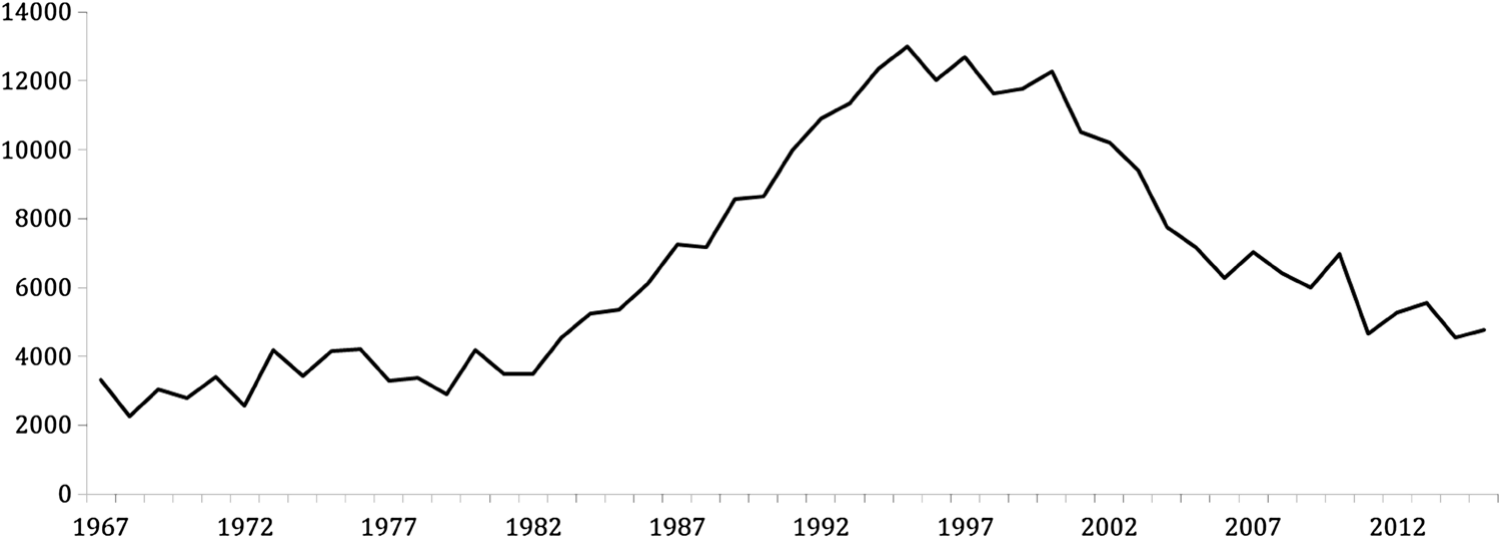
The winter mean number of Greenland white-fronted geese on Islay 1967–2015 (WWT and SNH count records)

The average numbers of Greenland white-fronted geese wintering on Islay increased from 3000–4000 individuals in the early 1980s (Fig. 3; Fox et al. 1998). From a peak of 15 500 attained in winter 1998/99, there has been a decline to a low of just over 4500 geese in 2011/12 (Fox and Francis 1999; Fox et al. 2012). Greenland white-fronted geese are legally protected from shooting throughout most of their range (with the exception of Wales where a voluntary ban is in place).

Up to 2500 breeding greylag geese *Anser anser* now also occur on Islay, having increased from a small number (fewer than 50) breeding on offshore islands in the early 1990s. Up to 1000 remain on Islay throughout the winter. Additionally, Canada geese *Branta canadensis* are present, but in very low numbers (fewer than 100 birds). Both species are legal quarry in the open season; Canada geese can be shot throughout the year under a general licence, and licences are frequently issued in the closed season for greylag geese to protect crops. Icelandic greylag geese are not known to use Islay, with no apparent influx of migrant greylags in winter. Greylags and Canada geese are therefore not of conservation concern on Islay and are controllable through existing mechanisms, so are not discussed further.

## Historical goose management on Islay

Mention of geese causing damage to grass on Islay and other islands was first made in the late 1800s (Harvie-Brown and Buckley 1892). Concerns about rising numbers were raised again in the 1960s when it was reported that barnacle geese grazing on improved grassland were causing damage to valuable crops (Patton undated). Whilst discussions took place between farmers and conservation organisations regarding limiting goose numbers, no action was taken at that time. A timeline highlighting the main events from this time onwards is presented in Table 1.

**Table 1:** Summary timeline of the main events in the development of Islay goose policy

Year	Conservation and monitoring	Goose management conflicts and actions on Islay	National (Scottish) legislation and policy development
1959	Start of regular barnacle goose census		
1960s		First concerns about impacts of geese on crops on Islay documented. No action taken	
1981			Wildlife & Countryside Act 1981 which came into effect in September 1992
1982	Commencement of whole-island counts on Islay		
1983		First management agreements with farmers	SSSI re-notifications
1984	RSPB acquire Loch Gruinart Reserve		
Mid-1980s			EC agri-environment schemes supported land improvement on Islay. Increase in area and quality of agricultural grassland
1987		Initial management scheme using scarers to scare geese into feeding areas	
1988			SPA classifications
1992	Commencement of field by field counts to support goose management scheme	Introduction of first whole-island goose management scheme with payments to farmers for feeding geese	
1999			Establishment of National Goose Forum with key stakeholders
2000		Setting up of Local Goose Management Review Group. Introduction of new goose management scheme to deliver national goose policy objectives including feeding geese but protecting crops by non-lethal scaring and licenced shooting to protect the most valuable crops	Introduction of National Goose Policy Framework and the setting up of the National Goose Management Review Group (and Goose Science Advisory subgroup) to advise Scottish Government
2005			Interim review of National Goose policy Framework
2009	SNH convene a Greenland white-fronted goose international workshop on Islay to develop an AEWA action plan		
2012	Greenland white-fronted goose flyway plan agreed AEWA (MoP5)		Review of National Goose Policy Framework
2014	Research by WWT to better understand behaviour of Greenland white-fronted geese on Islay (2 year project)	Existing goose management scheme amended to include elements to deliver the Islay Strategy	Development of Islay Sustainable Goose Management Strategy (using adaptive management approach)
2015	Collect baseline data for delivery of the Islay Strategy		

A decline in barnacle goose numbers from the mid-1970s to the early 1980s followed (Fig. 2), probably due to an increase in crop protection and sport shooting combined with some poor breeding seasons. The subsequent rise in numbers of both barnacle and Greenland white-fronted geese from the 1980s has been attributed to protection of geese through the introduction of the Wildlife and Countryside Act 1981. There was also an increase in the area of improved grassland on Islay, some of which was supported by European funding programmes from the 1980s such as the Agricultural Development Programme. Whilst the aim of these programmes was to benefit agricultural production, they also provided increased feeding opportunities for geese (The Scottish Office 1996).

**Fig. 4:**
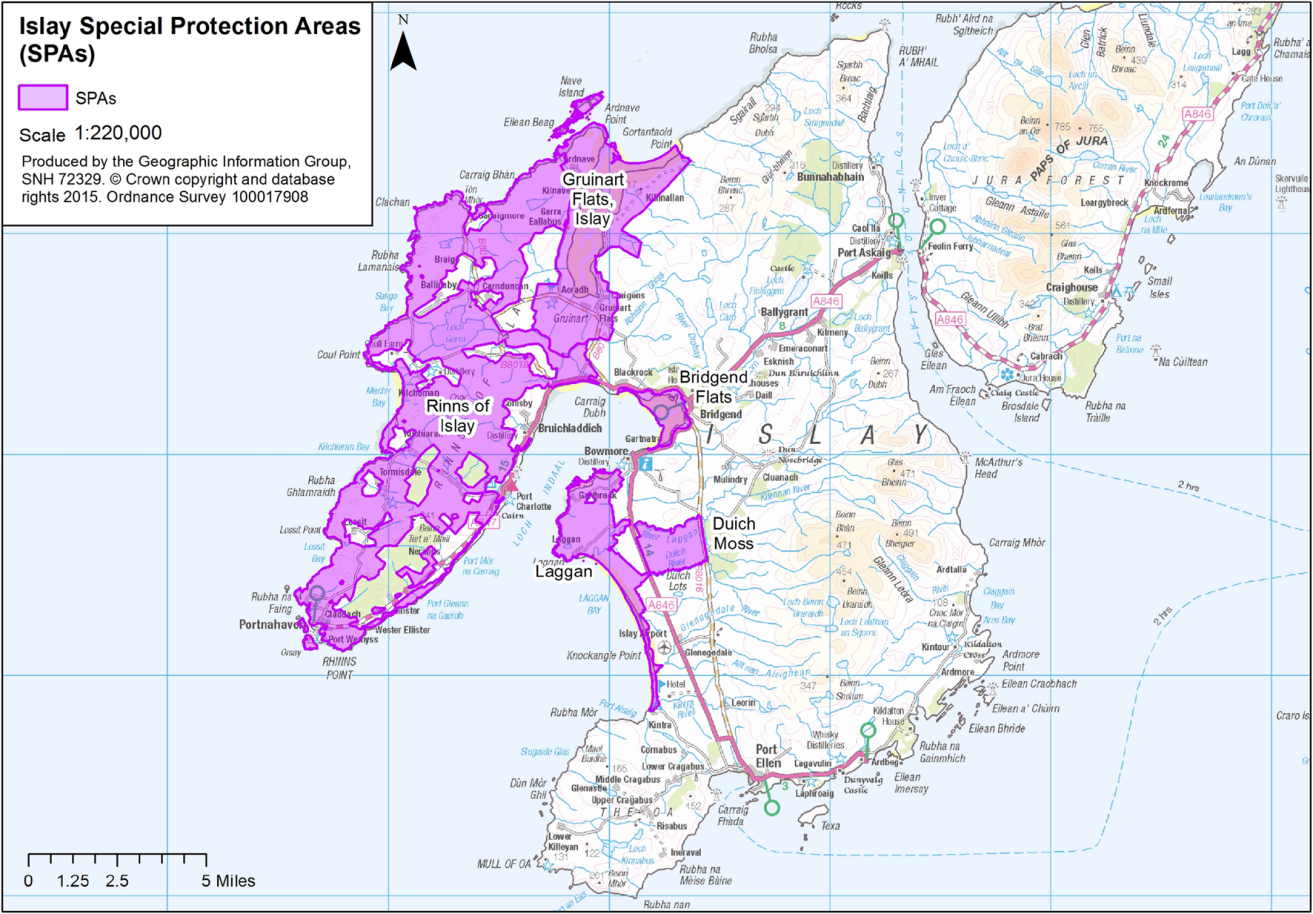
Map of the Special Protection Areas on Islay which are classified in accordance with the EC Birds Directive for geese

Whole-island goose counts down to individual farm scale were carried out twice a month from 1982, which was critical to underpinning island-wide goose policy through the 1980s and early 1990s. Re-notification of the three main Sites of Special Scientific Interest (SSSIs) under new legislation in 1983 allowed the development of the first goose management agreements and established the sanctuary management policy (Bignal et al. 1991). In 1984, The Royal Society for the Protection of Birds (RSPB) purchased Gruinart Farm on Islay. The aim of this was to maintain a refuge for barnacle geese on just under 900 ha of land around the main Loch Gruinart roost site (with Loch Indaal likely the core of the traditional distribution in Islay) and to reduce impacts of goose grazing on commercial farms in other parts of Islay (Bignal et al. 1991). The UK government’s conservation advisors at the time, the Nature Conservancy Council (NCC), classified five Special Protection Areas (SPAs) for geese (and other bird species) on Islay in 1988 (Fig. 4). These cover just under 15 000 ha, which is almost a quarter of the island, but are mostly in roosting areas; so large areas of agricultural grassland habitat used by foraging geese are not included within the SPAs.

### First management schemes

NCC was responsible for the introduction of the first management schemes, set up in the 1980s. These involved farmers within barnacle goose SPAs agreeing to maintain good quality grassland for grazing geese in return for management agreement payments to cover financial losses. Outside of the SPAs, a scaring scheme was set up in 1987 which used human scaring to try to displace geese from these areas into the SPAs (Bignal et al. 1991; Percival et al. 1997).

As overall numbers of geese continued to increase into the early 1990s, it became clear that the RSPB reserve and SPA refuge areas were supporting a smaller proportion of the geese over-wintering on Islay and that farmers outside of these areas were reporting increasing economic damage to their businesses as a result of goose grazing. The numbers of geese recorded outside of SPAs and reserves had increased, and farmers without management agreements argued that they were suffering similar economic impacts to areas with these. Whilst scaring did reduce geese on disturbed areas by just over 50%, the costs were uneconomic and some geese continued to use these areas (Percival et al. 1997). The scaring scheme was therefore not perceived to be sufficiently effective.

The first whole-island goose management scheme was therefore set up in 1992 by Scottish Natural Heritage (SNH), which replaced NCC. This involved paying farmers to allow geese to feed on grass without disturbance; all farmers on Islay were eligible to join, and payments were made on a per goose basis. At this time, SNH introduced regular systematic counts at an individual field scale which continue to this day; these record the number of geese (all species) present on Islay and on each farm unit. This scheme delivered mixed results. All farmers were able to claim payments for geese on their ground, but there was an ongoing debate about levels of payments, which farmers felt did not cover the costs of feeding geese. This was essentially a feeding scheme, and no scaring management was undertaken. Numbers of barnacle geese continued to grow throughout the 1990s, and peaked for Greenland white-fronted geese in 1995, declining thereafter (Figs. 2, 3).

### A new policy approach

After many years of conflict and various attempts at different approaches to resolve this, a new policy approach was initiated in 1999 by government to involve key stakeholders in goose management decision making and delivery at both local and national levels. This was a major step forward in providing an inclusive and empowering forum where the complex conflict could be discussed and resolved. Stakeholders included farming, conservation, land management, sporting and government interests, and formed a national group to review goose policy across Scotland and make recommendations on how goose management should be taken forward (The Scottish Executive 2000). These included the setting up of goose schemes in specific parts of Scotland, including Islay, to deliver an agreed set of policy objectives (The Scottish Executive 2000). Since then, goose management schemes on Islay have been developed and delivered by the Islay Local Goose Management Group (ILGMG) and co-ordinated at a national level by the National Goose Management Review Group (NGMRG), with advice from its subgroup, the Goose Science Advisory Group (GSAG) (see Bainbridge 2017).

The schemes on Islay and elsewhere in Scotland aim to deliver the following set of objectives agreed at a national level:

- Meet the UK’s nature conservation obligations for geese, within the context of wider biodiversity objectives;
- Minimise economic losses experienced by farmers and crofters as a result of the presence of geese; and
- Maximise the value for money of public expenditure (The Scottish Executive 2000).

This policy framework led to a new scheme being developed and launched by the ILGMG in autumn 2000. It recognised that farmers suffer economic losses as a result of geese grazing in high densities and made compensation payments for allowing geese to graze on parts of their farms. For the first time in an Islay scheme, farmers were also able to protect some parts of their grassland through scaring geese, and in certain situations, limited shooting of barnacle geese was licenced using the derogation under Article 9 of the EC Birds Directive. Payments made were for losses attributed to goose grazing, including costs of increased reseeding frequency and delayed turnout of livestock in the spring. The density of geese present on individual farms was taken into account within a payment calculation which was developed by the ILGMG (i.e. including farmer representatives themselves) and approved nationally by the NGMRG; farms which supported the highest densities of geese received a higher level of compensation. Payments made to farmers at this stage were the calculated costs of goose damage of approximately €700 000 year^−1^.

Whilst scheme funding is intended to cover costs of goose damage, it does not prevent damage occurring, and management activities to scare geese since 2000 have not been very successful in protecting crops. A number of different scaring techniques and devices have been used on Islay over a long period of time; a more detailed summary of the effectiveness of these can be found in McKenzie (2014). Whilst this effectiveness has never been fully evaluated on Islay, there is a significant amount of feedback from experienced farmers and goose scarers that suggest that these techniques are not fully effective. Bishop et al. (2003) reviewed literature on the effectiveness of bird scaring techniques in a number of locations and included a critique of some methods used on Islay. This paper broadly supports the anecdotal evidence available from Islay and elsewhere that habituation is the main factor behind ineffectiveness.

### Reviews of goose management costs

National reviews of goose management costs conducted by NGMRG in 2005 and 2008 then resulted in payments to Islay farmers increasing as a result of increasing goose densities and farming costs (fertiliser, fodder and fuel). Following both reviews, the Scottish Government decided that costs could not be fully supported to reduce overall costs to the taxpayer, and so the budget offered to farmers was less than the calculated cost of supporting geese. Further cuts to payments were made following a review of all goose schemes and national goose policy in 2010 (Crabtree et al. 2010). At this stage, the Scottish Government determined that scheme costs needed to be limited, payments should be focused on agricultural activities delivering most benefits for geese and financial intervention should be targeted at species with the highest conservation status. Alternative mechanisms for managing protected species that were no longer of the highest conservation status were explored. In the case of Islay, this meant that Greenland white-fronted geese were the focus of conservation intervention and alternative mechanisms for managing barnacle geese were explored.

The overall budget for goose management in Scotland in 2011/12 was reduced, resulting in a reduction of the Islay scheme budget from €1.06 m in 2010/2011 to €833 000. The scheme was accordingly revised by the ILGMG to include, amongst other measures, weighted payments towards supporting Greenland white-fronted geese. This revision was not thought by farmers to be successful as it did not appear to reduce damage caused by barnacle geese (of which farmers still had to support large numbers), and it did not halt the decline in Greenland white-fronted geese numbers. It did not therefore achieve the stated objectives of minimising economic losses to farmers and meeting international conservation obligations. Accordingly, the weighted payments were dropped.

NGMRG and all local goose management groups then again reviewed the payment calculations prior to new three-year schemes set up from 2012 to 2015. The Islay scheme review included updated costs for individual elements of the payment calculation and rationalised elements paid for. The increase in costs of fuel, fertiliser and fodder since the 2008 payment review, along with using a longer term (from 3 to 7 years to better reflect the average costs to farmers working with a 7-year rotational grassland management system) average goose count to calculate individual farm payments meant the calculated cost of the Islay scheme rose to approximately €1.9 m.

Scottish Government could not commit that level of funding due to budget constraints, and so funding offered for the 2012/13 scheme was just c. €1 m. The ILGMG accepted this on condition that SNH and Scottish Government undertook to consider reducing barnacle goose numbers to a level which reduced damage on Islay farms. Concern remained that the level of damage was having a serious impact on farm businesses and that, if it continued, there was a long-term threat to these.

In late 2012 and following engagement with local farmers, there was Ministerial level involvement in establishing a commitment to adaptive harvest management. SNH and Scottish Government therefore agreed to develop a long-term sustainable management strategy for geese on Islay using an adaptive management approach, and a project Steering Group was set up which additionally included representatives of the Islay NFUS. The aim of the resulting Islay Sustainable Goose Management Project was to develop the 10 year Islay Sustainable Goose Management Strategy (hereafter, the Strategy) for management of all species of geese on Islay. This formed the basis of a new Islay Goose Management Scheme to be delivered by the ILGMG, which was launched in autumn 2015.

## Islay sustainable goose management strategy (2014–2024)

The Strategy (McKenzie 2014) was developed with an ongoing process of stakeholder engagement (government, conservation NGOs, farmers and land managers, as well as Scottish Government consultation with other countries in the range of the Annex 1 geese) and proposes to follow adaptive management principles. It seeks to address the national goose policy objectives on Islay and also considers how this will impact on other interests such as tourism and non-wildfowl sporting interests.

To do this, the Strategy seeks to

- develop habitat management techniques to support feeding of Greenland white-fronted geese through provision of diversionary feeding and management of *Juncus* rush pasture;
- ensure that large areas of suitable habitat are available to all species of geese as undisturbed roosting and feeding areas;
- maintain a viable number of barnacle geese at a level which meets conservation obligations;
- ensure that there will be no adverse effect on site integrity of the Special Protection Areas by considering the conservation objectives of those sites;
- reduce damage to grass crops by reducing the number of barnacle geese on Islay, and therefore reducing the impact of geese on the agricultural economy; and
- ensure that compensation payments to farmers for goose damage are targeted at the most appropriate management activities (i.e. those growing grass).

Broadly, the Strategy is two-fold. The first part aims to significantly reduce agricultural damage on Islay, by reducing barnacle goose numbers feeding on grass crops, and by continuing research into developing new crop protection and scaring methods. It accepts that the current levels of goose grazing on Islay causes significant damage to agricultural land and has an economic impact on livestock producers (which is why goose schemes have been providing compensation for goose damage across Islay to farmers since 1992 and within key sites since the mid-1980s). Compensation available to farmers for damage caused does not currently meet the total costs of supporting goose numbers, and scaring is not effective enough in reducing agricultural damage. The barnacle goose numbers will be maintained at a level which continues to meet conservation obligations on Islay and across the international range. That level will also ensure that the spectacle of thousands of wintering geese, enjoyed by many tourists to Islay, is maintained. The second part aims to manage Greenland white-fronted geese to increase numbers to a level which restores their numbers on individual SPAs on Islay, and which makes a positive contribution to restoring the international favourable conservation status of these geese. The aim is to minimise disturbance to Greenland white-fronted geese and to develop trials to provide better feeding opportunities away from high-value agricultural grass crops.

## Legal basis for shooting barnacle geese

Any reduction in barnacle goose numbers requires the issue of licences by SNH to kill birds to prevent serious agricultural damage. Licences are issued for this purpose only if SNH are content that there are no other satisfactory solutions and they meet the provisions set out in the derogation under Article 9 of the EC Birds Directive. The tests which need to be applied prior to licences being issued can be summarised as follows:

1. Is serious damage being, or is likely to be, caused by geese at the site?
2. Have all other reasonable non-lethal scaring measures both been tried and found to be ineffective; or are impracticable; or are unlikely to work at the site?
3. Is it reasonable to consider that shooting geese will reduce, or prevent from increasing, the level of damage (whether through scaring or direct reduction of numbers)?

With regard to the first point, it is not always possible to define levels of damage in simple quantitative terms, but the absence of clear data does not mean that damage is not occurring nor is not serious. Guidance issued from the EC on the use of Article 9 derogations to manage great cormorants *Phalacrocorax carbo* provides details on how a pragmatic approach to the problem might be applied as follows:

“In all cases, the concept of ‘serious damage’ as used in the Birds Directive, and interpreted on the basis of the EC guidance on damage by great cormorants to fisheries involves the following:

- Firstly, it clearly relates to economic damage to fisheries and/or also economic damage to fisheries-related recreational interests. The concept of ‘damage to fisheries’ is clearly related to the economy of turnovers and expected profits;
- Secondly, derogations issued under Article 9 of the Birds Directive are intended to prevent serious damage; therefore it is not only a response to already proven damage but also to the strong likelihood that this will take place in the absence of action. But, the chance that damage might occur does not suffice as, if damage is not yet evident, past experience should demonstrate a high probability of its future occurrence;
- Thirdly, there must be a basis for concluding that damage will be serious in the absence of action.” (European Commission 2013).

In considering these points in relation to goose management, numerous studies have been commissioned by farmers, academics and nature conservation organisations to try to quantify the level and cost of damage caused by geese on Islay. These studies have demonstrated that geese do cause agricultural damage on Islay, and calculations of the economic costs of that damage have been made and refined over the past 20–25 years [a summary can be found in the Strategy (McKenzie 2014)].

With regard to the second point, non-lethal and lethal scaring have taken place, but significant damage to productive grassland still occurs. Money does provide some level of compensation, but the continuing level of goose grazing acts on the ability of farmers to produce grass and carry profitable levels of stock in the longer term. As such, compensation cannot guarantee that agricultural activity in its current form will continue. For these reasons, scaring (including lethal scaring at levels prior to the Strategy) is not considered to be a satisfactory alternative to reducing barnacle goose numbers, as it does not prevent serious agricultural damage. Therefore, the Strategy concluded that the conditions for using the derogation have been met.

## Management to benefit greenland white-fronted geese on Islay

We do not fully understand the reasons for the decline in Islay of Greenland white-fronted goose numbers, but it does mirror a decline in the global population. This is thought to be due to poor breeding success as a result of either changed climatic conditions and/or competitive interactions with Canada geese on the breeding grounds (Boyd and Fox 2008; Stroud et al. 2012; Weegman et al. 2016). To help inform Strategy implementation, ongoing research undertaken by SNH and the Wildfowl and Wetlands Trust (WWT) is attempting to better understand Greenland white-fronted goose habitat use, movements and effects of disturbance on Islay which will feed into refinement of management activities in future.

There are limited options within current agri-environment programmes to support management for Greenland white-fronted geese, mainly through management of rush pasture to improve feeding opportunities. During the winters of 2013/2014 and 2014/2015, trials of rush pasture management and diversionary feeding were therefore established. Initially this was carried out experimentally on ground managed by RSPB and farmer volunteers. The results of this work are still being analysed, but it is hoped that standard techniques can be established so that good quality feeding on traditional habitats (such as wet grassland and peatland) and supplementary feeding can be provided to Greenland white-fronted geese to off-set any potential impacts of scaring of barnacle geese. In future years of the Strategy, this management will be encouraged and supported in key areas either through further research (if required), a goose scheme or through agri-environment schemes.

## Islay local goose management scheme

The Strategy is delivered through the current (2015) Islay Local Goose Management Scheme (the Scheme). In previous years, around 6000–7000 ha of productive farm land (including improved and unimproved grassland, dunes, machair and saltmarsh) was included within the scheme, and it is expected that the area will remain fairly constant over the next 5–10 years. Scaring, including shooting, can take place on around 15–20% of the land whilst the remainder constitutes undisturbed feeding area. To deliver the conservation aims of the Strategy, provision continues to be made for significant areas of undisturbed feeding and refuge areas for both barnacle and Greenland white-fronted geese on all farms. These refuge areas include improved and permanent pasture, dune grassland and saltmarsh, and comprise at least 70% of the available grassland habitats on which geese feed. However, there is an ongoing commitment for payments to farmers for management of those areas where significant damage occurs. Provision of goose grazing on grass pastures is still detrimental to farm businesses, but with fewer geese being supported as a result of reduced barnacle goose numbers, it is hoped that damage levels should be concomitantly reduced.

Management to reduce barnacle goose numbers will be carried out under the Scheme, with farmers and SNH contractors carrying out the shooting under strictly controlled licencing. The total bag limit for the island has been carefully calculated using the most recent Islay population viability analysis (PVA) (Trinder 2014). The PVA used historical demographic data (1995–2011) to predict future population trends given different levels of harvest pressure. An Excel model version of the PVA is used to calculate bags with current counts, but as demographic rates vary, an additional simple Excel model is used to sense-check predictions with other current data, and a precautionary 10% level of crippling loss is assumed and accounted for. This approach has been endorsed by the GSAG. The total bag as calculated with the PVA is then released in two phases by SNH, with the second only released once the December counts are known and only if they are as high as expected. Updates to the PVA are planned to take account of more recent demographic data. The total released bag is then apportioned to participating individual farmers using average goose density data on each farm unit (any unused bags are redistributed). Until 2014/2015, bag limits had been set to try to maintain barnacle goose numbers, but from 2015/2016, bag limits have been increased slightly to begin to lower numbers gradually over a 10-year period.

It is recognised that killing or scaring barnacle geese may have indirect impacts upon Greenland white-fronted geese in some locations. Therefore the Scheme restricts shooting to limited locations, usually the youngest grass swards, and it prevents shooting of barnacle geese if Greenland white-fronted geese are present in a mixed flock. Management activities within the Scheme which potentially impact upon SPAs have undergone a Habitats Regulation Appraisal (HRA) to comply with Article 6 of the Habitats Directive to confirm that there will be no adverse effects on the integrity of individual SPAs (SNH 2014).

## Quantifying damage

One of the main aims of the Strategy is to reduce the level of damage suffered by farm businesses as a result of goose grazing. Whilst it is not yet known if goose numbers have a linear relationship with the amount of damage caused, this assumption is a starting point and the Strategy takes an adaptive management approach to test this. Barnacle goose numbers on Islay will be reduced in increments with levels of damage monitored throughout the 10-year period. Damage is being measured in the short term by sward height monitoring on sample fields and in the longer term by the frequency of reseeding.

In the first year of the Strategy (2014/2015), the baseline level of damage was measured. Based on GSAG advice, this was done by installing five exclosure cages in each of 26 stock-free third-year reseed grass fields and measuring grass growth in protected and non-protected areas of these fields. Third-year fields were chosen as the best way to measure damage against a decreasing number of geese; levels of scaring on first- and second-year fields are likely to remain high even with fewer geese and so were not considered useful options. Relative grazing levels by geese and various mammalian herbivores were measured through dropping counts, so that damage could be apportioned to the different herbivores and thus measure the level of damage specifically caused by geese. This type of monitoring will continue, initially annually, for the period of the Strategy to ensure that the level of damage caused by geese is measured and that the impacts of reducing goose numbers can be demonstrated. Attempts to determine dry matter grass production in the winter of 2015 identified methodological problems, but results from the exclosures suggested that grass height within plots protected from any grazing was higher than plots exposed to grazing, that this difference was mainly due to grazing by barnacle geese and that this effect continued to be demonstrable into May, just prior to silage being cut.

Economic losses will be monitored using the payment rate calculation, which gives an assessment of the full costs of damage. The payment rate calculation considers all elements where farmers incur additional costs as a result of goose damage and has been agreed by NGMRG and ILGMG. This rate is applied to the relevant habitat and goose density on the individual farm and used to calculate the compensation payment to each farm unit.

The aim is to reduce damage by 15–20% in the first half of the Strategy (i.e. by 2019) by reducing the barnacle goose numbers by the same proportion (discussed below). Measurements of sward height will be taken every year, but an assessment of how well the reduction in numbers meets the aims will be taken after several years of monitoring. If the monitoring can demonstrate that the actions taken to reduce barnacle numbers are reducing the levels of damage by a similar level, then the aim is to achieve a further reduction in damage of 10–15% by the end of the Strategy period (2024). The aim is for the total reduction in goose damage by the end of the Strategy to be 25–35% of that of the baseline. These percentages were thought to be pragmatically achievable, as well meeting political and conservation commitments. If actions to reduce damage by 2019 cannot be demonstrated, then the Strategy and management actions will be reviewed. This is similar to the approach advocated by other countries which are attempting to develop management plans for protected geese (including Annex 1 species), for example The Netherlands are setting targets based on goose numbers to try to reduce damage to the level that it was in 2005 (Wadden Sea Forum Goose Management Group 2013).

## Setting a lower threshold level of barnacle goose numbers

In considering the reduction in damage and linking that to a reduction in barnacle goose numbers, the Strategy looked at a number of ways to set threshold levels which ensure that all the goose policy objectives are met. At the higher end of the scale, barnacle geese should not be allowed to increase beyond numbers at the start of the Strategy (41 250 ± 10%). At the lower end of the scale, a minimum ‘safe’ threshold level was calculated considering the following:

### Biological perspective

SNH ornithologists used the current PVA (Trinder 2014) to calculate that the minimum ‘safe’ level is 23 100 birds (i.e. if levels of shooting in Iceland remained at current levels, we could be 97.5% confident that if numbers on Islay remained above c. 23 100, they would not decline further so long as shooting on Islay then ceased; Urquhart 2014). Allowing ±10% for count variation (a percentage which generally covers the count fluctuations we see), this translates to a range of 20 790–25 410 birds.

### Practical perspective

The lower threshold level needed to be practically achievable with available resources.

### Special Protection Area (Natura) perspective

Islay barnacle goose numbers at the time of SPA classifications in 1988 were c. 20 000 (citations available from SNH Sitelink). To meet international obligations, we are expected to maintain or restore barnacle goose numbers at individual SPA level, so the range proposed within the Strategy needed to ensure that barnacle goose numbers on Islay remain comfortably higher than 1988 levels. The range suggested under the biological perspective was therefore not considered to be high enough.

Article 2 of the Birds Directive relates to the maintenance of bird populations across their natural range. Whilst the growth in Islay barnacle numbers appears to have stabilised, numbers across the rest of the winter range have continued to increase in recent years (Mitchell and Hall 2013). Monitoring of the wintering Greenland barnacle goose population will continue through the five yearly census.

Taking these considerations into account, the Strategy therefore proposes that the lower threshold range on Islay should lie between 28 000 and 31 000 barnacle geese. To achieve the proposed reduction in damage, at the start of the Strategy, the PVA (Trinder 2014) suggested that numbers would need to be reduced by around 2000–2500 birds per annum for 8–10 years, which is not markedly higher than previous lethal scaring levels of shooting (e.g. between 2012/13 and 2014/15, the average number shot under licence was 1745, which was on average 4.1% of the mean season number). To shoot 2000–2500 geese annually is thought to be achievable with additional resources and flexibility in the way shooting is organised. Shooting is carried out under licence and will only be carried out for the purpose of preventing serious damage to crops.

Work to reduce barnacle goose numbers on Islay towards the lower threshold range began in 2015. At the same time, farmer scaring continued using non-lethal methods to provide short-term protection of crops in some locations. Although it has been difficult to date to rigorously test for differences in effectiveness between lethal and non-lethal scaring techniques (Douglas et al. 2009), it is possible that higher levels of lethal shooting may reinforce the effectiveness of non-lethal methods. Reduced competition for grazing by a lower density of geese may also increase effectiveness of non-lethal scaring, e.g. if there is sufficient grass resource in the feeding areas to support a certain level of goose grazing, then there could be less likelihood of geese trying to graze on scaring areas.

As the numbers are reduced, monitoring of the impact of that reduction will continue. If this demonstrates that an agreed outcome is reached (e.g. where measured damage is reduced and the actions taken reduces economic losses to farmers), shooting to reduce the population will cease prior to reaching the lower threshold level. If there is a sudden increase in numbers on Islay which exceeds the acceptable upper threshold and there is no evidence that these birds have come from other parts of the range, the bag limit may be increased accordingly. If there is a significantly larger than predicted decrease in numbers, the bag limit will be reduced or shooting halted until they stabilise. The situation in other countries in the range will also be monitored through discussion with international counterparts, and, if the number of birds killed in Iceland changes significantly (data updated annually on the Statistics Iceland website), targets on Islay will be adjusted to ensure that actions there have only the intended impacts, i.e. to ensure that there are not unintended cumulative impacts with management practices elsewhere in the range. Once a level has been reached which satisfactorily minimises the damage to crops (at or above the minimum threshold level), there will be ongoing shooting, to maintain barnacle numbers at that agreed level to protect crops.

## Challenges of adaptive management

The key factor in developing an adaptive management Strategy for Islay geese was increasing barnacle goose numbers and indications from the PVA that these were likely to continue increasing, resulting in ever increasing pressures on farmers suffering goose damage. However, as the Strategy has been developed and introduced, we have seen a stabilisation of the trend. Numbers over the past few years have fluctuated from a winter seasonal average (of the counts in November, December, January and March) of almost 47 000 in 2012, to 38 000 in 2014/2015 (although this was likely due to an undercount in November 2014) and 43 000 in 2015/2016. The grass damage measurements are practically constrained by the number of appropriate third-year reseed fields, and it may ultimately be difficult to relate changes in damage levels to numbers of geese. There is variation associated with environmental factors that affect grass growth, and the overall maximum possible decrease in goose numbers is relatively small, given that baseline measurements were made when goose numbers were considerably lower than the 47 000 birds of 2012 when planning began.

Costs of delivering the Strategy are likely to include payments to farmers to compensate for some of the damage done by geese (currently in the region of €1.07 m) and will additionally need to cover employment of marksmen (to ensure SNH retain control over barnacle goose shooting); regular goose counts and age assessments; monitoring work to measure the damage caused by geese and any reduction in that damage as a result of fewer geese; research to develop diversionary feeding techniques; and trials of any new scaring techniques. Delivery of the 10-year Strategy via the Scheme will require a secure budget throughout, but this is challenging. Government funding settlements are generally for much shorter periods of time given that their budgets are set based on affordability and the range of other priorities at the time.

The adoption of an adaptive management approach requires that good quality data continue to be collected to inform both the population modelling and decision making processes. Whilst the Strategy is being implemented with the best available information, it is also key that research is continued to investigate currently unclear aspects of the system. SNH are therefore supporting a Scotland-wide barnacle goose ringing programme to clarify contemporary movement links with other islands, and ongoing work to understand the causes of decline and possible management remedies for Greenland white-fronted geese.

Finally, the involvement of key stakeholders in the development of the Strategy has worked well via local and national groups, and formal and informal consultations. This has resulted in useful input at the development stage and a good understanding of the aims of the Strategy by all those involved. Ongoing clear lines of communication through local and national meetings will be vital to ensuring that there is useful stakeholder input in the longer term. However, not all stakeholders have agreed with the approach taken to date, and the conservation NGOs have raised a complaint with the EC about the Strategy (which is still ongoing) and have resigned from the NGMRG and subgroups. There is a risk that complaints may result in a change of approach in future.
